# Long-term results of additional pulmonary blood flow with bidirectional cavopulmonary shunt

**DOI:** 10.1186/s13019-020-01335-4

**Published:** 2020-09-29

**Authors:** Ryosuke Kowatari, Yasuyuki Suzuki, Kazuyuki Daitoku, Ikuo Fukuda

**Affiliations:** 1 Department of Thoracic and Cardiovascular Surgery, Hirosaki University School of Medicine, 5 Zaifucho, Hirosaki, Aomori, 036-8562 Japan; 2 Department of Cardiovascular Surgery, Ibaraki Clinical Education and Training Center, University of Tsukuba Hospital, Tsukuba, Ibaraki, 305-8576, Japan

**Keywords:** Fontan, Additional pulmonary blood flow, Late complication, Liver function

## Abstract

**Objective:**

We evaluated additional pulmonary blood flow at the time of bidirectional cavopulmonary shunt and its effects on the Fontan procedure and long-term outcome of Fontan circulation and liver function.

**Methods:**

We included 22 patients (16 boys, 6 girls) having undergone bidirectional cavopulmonary shunt with additional pulmonary blood flow between April 2002 and January 2016. Mean age and body weight were 20 ± 13 months and 7.5 ± 6.5 kg, respectively. We retrospectively evaluated the patients’ clinical data, including cardiac catheterization data, liver function, and liver fibrosis markers.

**Results:**

All patients were alive with a New York Heart Association status of I at the long-term follow-up. Changes between pre-bidirectional cavopulmonary shunt and 101 months after the Fontan procedure included the following: the cardiothoracic ratio of chest X-ray decreased from 52.2 ± 3.9% to 41.8 ± 5.9% (*p* < 0.001); systemic ventricle end-diastolic pressure decreased from 11.4 ± 3.2 mmHg to 6.9 ± 3.6 mmHg (*p* < 0.001); and the pulmonary artery index decreased from 485.1 ± 272.3 to 269.5 ± 100.5 (*p* = 0.02). Type IV collagen, hyaluronic acid, and procollagen levels increased over the normal range 116 months after the Fontan procedure.

**Conclusions:**

The additional pulmonary blood flow at the time of bidirectional cavopulmonary shunt may contribute to pulmonary arterial growth at the Fontan procedure with low pulmonary arterial resistance and without ventricle volume overload. The Fontan circulation was well-maintained at the long-term follow-up, while liver fibrosis markers were above their normal values.

## Backgrounds

The bidirectional cavopulmonary shunt (BCPS) is a palliative step in the staged Fontan procedure for patients with a functional univentricular heart [1, 2]. Studies have revealed that the preserved additional pulmonary blood flow (APBF) promotes interstage pulmonary artery (PA) growth and increases oxygen saturation in patients after BCPS [3, 4]. Moreover, APBF could preclude the arteriovenous fistula development in the lungs by exposure to hepatic venous flow [5]. Conversely, BCPS with APBF might prolong pleural effusion and increase ventricular volume overload, leading to the potential deterioration of atrioventricular valve regurgitation [6]. These short-term outcomes have been well discussed, whereas little is known about long-term outcomes in patients who underwent BCPS with APBF. Thus, whether APBF should be preserved at the time of BCPS remains controversial [7–10].

Liver complications including fibrosis, cirrhosis, and hepatic cell carcinoma, which are the major late complications after the Fontan procedure, have recently received considerable attention [11–14]. Hepatic dysfunction after the Fontan procedure is caused by passive venous congestion of the liver because of higher-than-normal central venous pressure (CVP). Furthermore, the contribution of BCPS with APBF toward liver dysfunction after the Fontan procedure remains unclear.

Here, we evaluated the efficacy of the preserved APBF on the Fontan procedure and report the long-term follow-up results, including those of Fontan circulation and liver function, in patients who underwent BCPS with APBF.

## Methods

### Patients

We enrolled 22 patients (16 boys and 6 girls) of the 23 patients who underwent BCPS with APBF at the Hirosaki University Hospital between April 2002 and January 2016. One patient was excluded from this study because he developed hypoxic encephalopathy soon after birth at another hospital, and his family denied consent to the Fontan procedure. The institutional review board of Hirosaki University approved this study. The mean age and body weight were 20.1 ± 13.1 months and 7.5 ± 6.5 kg at BCPS, respectively, 37.5 ± 12.9 months and 12.6 ± 2.2 kg at the Fontan procedure, respectively. Seven and 15 patients had right dominant and left dominant ventricular types, respectively. Four patients had heterotaxy syndrome. The patient characteristics are listed in Supplementary Table 1.

### Surgical management

Systemic-to-pulmonary (SP) shunt or pulmonary artery banding (PAB) was performed according to individual requirement prior to BCPS. In pulmonary atresia cases, at least one SP shunt was placed opposite to the superior vena cava (SVC). At the time of on-pump-beating BCPS, cardiopulmonary bypass (CPB) was established via the aortic and SVC cannulation. For aortic clamp cases undergoing a concomitant procedure such as Damus–Kaye–Stansel anastomosis, atrial or inferior vena cava cannulation was added. CVP was monitored in all patients using a catheter placed in the SVC. We regulated APBF so that CVP did not exceed 18 mmHg soon after weaning from CPB at BCPS. In patients who underwent PAB prior to BCPS, the PAB tape was tightened. The length of the banding tape (mm) corresponded to twice the weight (kg) of the patient. When the patient underwent an SP shunt, the SP shunt was left open, without clipping or partial ligation. When CVP was > 18 mmHg with unstable circulation after CPB weaning, we decided to reduce the PA blood flow**.** At the time of the Fontan procedure, all APBF was removed.

### Data collection

Data were collected until December 2018. Operative and perioperative data including CVP, duration of chest tube drainage, and intubation time were collected by chart review. Cardiac catheterization was performed in all patients before and after BCPS within an interval of 14.9 ± 6.1 months. Cardiac catheterization was performed at 10.4 ± 11.6 months and 100.5 ± 32.7 months for 18 and 13 patients after the Fontan procedure, respectively. PA index (PAI), mean PA pressure (PAP), PA resistance (Rp), and systemic ventricular end-diastolic pressure (SVEDP) were obtained. The cardiothoracic ratio (CTR) of chest X-ray and saturation of percutaneous oxygen (SpO_2_) were also obtained for the same time. Brain natriuretic peptide (BNP) levels in the blood and liver fibrosis markers [aspartate aminotransferase (AST), alanine aminotransferase (ALT), gamma-glutamyl transpeptidase (γ-GTP), T-Bil, collagen type IV, hyaluronic acid, and procollagen] were measured at 1, 54.4 ± 18.9, and 116.4 ± 8.8 months after the Fontan procedure in 22, 13, and 8 patients, respectively.

### Statistical analysis

Statistical analysis was performed using the SPSS software, version 20 (IBM, Armonk, NY, USA). Continuous data are expressed as mean ± standard deviation or median (range). The catheter data, CTR, and SpO_2_ at pre-BCPS (baseline) were compared to those at other time points (pre-Fontan, post-Fontan, and long-term). The liver function and fibrosis markers between baseline and mid-to-long after the Fontan procedure were also compared. A Mann-Whitney *U* test was performed to assess difference, and statistical significance was defined as *p* < 0.05. Laboratory data were not statistically analyzed because of the insufficient number of patients.

## Results

### BCPS procedure

There was no operative or hospital death. Other perioperative data of the BCPS are shown in Table 1. CPB and operative durations were 42.5 ± 14.9 and 231.0 ± 91.4 min, respectively. The median duration of mechanical ventilation was 15.0 h (range, 1–121 h). The CVP just after BCPS completion was 16.7 ± 2.6 mmHg. In five patients, the CVP levels were approximately 18 mmHg just after CPB and decreased to < 15 mmHg in all patients within 12 h. There were no adverse events such as SVC syndrome, mediastinitis, and pleural or pericardial effusions requiring drainage procedures. In one patient with moderate common atrioventricular valve insufficiency, no exacerbation of valvar regurgitation was observed after BCPS. Major pulmonary arteriovenous malformations were not seen in any patient.

**Table 1:** Patient characteristics and perioperative data including the source of the additional pulmonary blood flow (APBF) at bidirectional cavopulmonary shunt (BCPS)

Age (months)	20 ± 13
Sex (men:women)	16:6
Body weight (kg)	7.5 ± 6.5
Operative time (min)	231 ± 91
CPB time (min)	42 ± 15
(Cardiac arrest: 3) Source of APBF	
SP shunt	
4 mm	10
3.5 mm	5
Antegrade PA flow	
Tightened PA 15–20 mm (median 18 mm)	6
Native PA without banding	1
Concomitant surgery (n)	4
Aortic plasty: 2, PAPVR repair: 1, PA plasty: 1	
CVP (mmHg)	16.7 ± 2.6
Intubation (h)	22 ± 30
ICU stay (days)	3.4 ± 1.7
Prolonged (> 2 weeks) pleural effusion (n)	0
SVC syndrome	0
Death	0

*CPB* Cardiopulmonary bypass, *SP* Systemic-to-pulmonary, *PA* Pulmonary artery, *PAPVR* Partial anomalous pulmonary vein return, *CVP* Central venous pressure, *ICU* Intensive care unit, *SVC* Superior vena cava

APBF was preserved until the Fontan procedure in all patients. The source and size of APBF are presented in Table 1. Fifteen patients underwent SP shunt as the first palliative procedure; the SP shunts were preserved in 13 patients without decreasing PA blood flow, one patient with pulmonary stenosis underwent SP shunt ligation, and one patient underwent SP shunt ligation with PAB (20-mm diameter). Five of the six patients who underwent PAB as the first palliative procedure underwent PAB again at BCPS, and one patient underwent left PAB because he had unbalanced pulmonary blood flow due to the migration of the banding tape, leading to an excessive flow in the left PA; the length of the banding tape was 15 mm.

### Fontan procedure

All patients underwent the Fontan procedure at a mean age of 37.5 ± 12.9 months and body weight of 12.6 ± 2.2 kg. No patient showed a drop-out or interstage death. The perioperative data of the Fontan procedure are presented in Table 2. The interval between BCPS and the Fontan procedure was 17.4 ± 6.7 months. The Fontan procedure type was extracardiac total cavopulmonary bypass in 20 patients and atriopulmonary connection in two patients. Fenestration was not performed in all patients. Concomitant surgeries were performed in six patients: three patients received PA plasty, one patient received atrioventricular valve plasty, one patient received Damus–Kaye–Stansel anastomosis, and one patient received ascending aorta replacement. CPB and operative durations were 107.8 ± 52.2 min and 339.9 ± 111.1 min, respectively. The duration of mechanical ventilation was 67 ± 94 h (median 16.0 h). The CVP soon after weaning from CPB during the Fontan procedure was 16.0 ± 3.1 mmHg. Nine patients received nitric oxide inhalation therapy. The duration of pleural drainage was 11.0 ± 7.7 days and prolonged (> 2 weeks) pleural effusion was seen in six patients. There were no adverse events; in particular, no Fontan circulation failure occurred. Warfarin was administered to all patients except one who underwent the Fontan procedure along with atriopulmonary connection. No patient developed any thrombotic or bleeding event during the follow-up.

**Table 2:** Perioperative data at Fontan procedure

Age (months)	37 ± 13
Body weight (kg)	12.6 ± 2.2
Fontan type (n)	
TCPC 16 mm	2
18 mm	18
APC	2
Operative time (min)	340 ± 111
CPB time (min)	108 ± 52
(cardiac arrest: 3, ventricular fibrillation: 6)	
Concomitant surgery (n)	6
(PA plasty: 3, AVV plasty: 1, DKS: 1, Asc. aortic replacement: 1)	
CVP (mmHg)	16 ± 3.1
Intubation (h)	67 ± 94
ICU stay (days)	5.8 ± 5.1
Prolonged (>2w) pleural effusion (n)	6
Fontan take down	0
Death	0

*TCPC* Total cavopulmonary connection, *APC* Atriopulmonary connection, *CPB* Cardiopulmonary bypass, *PA* Pulmonary artery, *AVV* Atrioventricular valve, *DKS* Damus–Kay–Stansel, *CVP* Central venous pressure, *ICU* Intensive care unit

### Post-Fontan course

There were no late deaths or any major adverse cardiac or cerebrovascular event. All patients were classified as New York Heart Association (NYHA) status I (no limitation of physical activity) at the mid-to-long-term follow-up. There were three reoperations after the Fontan procedure. One patient of right isomerism underwent common atrioventricular valve replacement with a 31-mm On-X valve (On-X Life Technologies, Austin, TX, USA) and catheter ablation through median sternotomy approach 6 years after the Fontan procedure. His atrioventricular valve regurgitation level was moderate. Operative findings showed that the main cause of AV regurgitation was hypoplasty of the atrioventricular valve, indicating that atrioventricular regurgitation will progress in the future. Therefore, we decided to perform atrioventricular valve replacement at the time of ablation. One patient underwent abnormal muscle band resection of the left ventricular outflow tract 6 years after the Fontan procedure. One patient underwent total cavopulmonary connection conversion using a 20-mm Gore-Tex graft (W. L. Gore & Associates, Newark, AZ) 10 years after the atriopulmonary connection Fontan. Two patients experienced intrapulmonary shunts that did not require any intervention.

### Clinical data

**Fig. 1:**
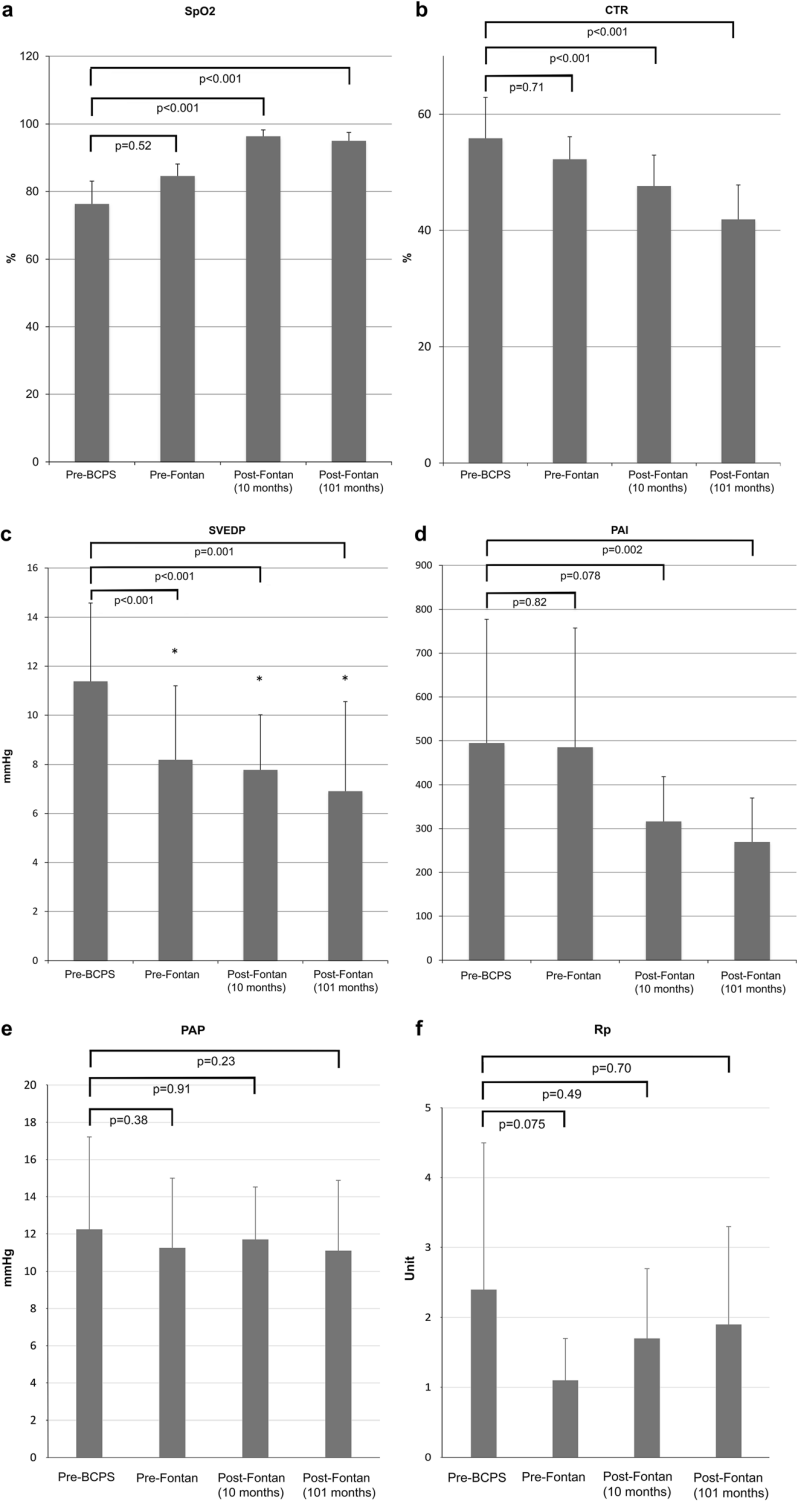
Changes in (a) systemic arterial oxygen saturation (SpO2), (b) cardiothoracic ratio of the chest X-ray, (c) systemic ventricular end-diastolic pressure (SVEDP), (d) pulmonary artery index (PAI), (e) mean pulmonary arterial pressure (PAP), and (f) pulmonary arterial resistance (Rp)from baseline before the bidirectional cavopulmonary shunt (BCPS) to an average of 101 months after the Fontan procedure

The data change between the pre-BCPS period (baseline) and approximately 100 months after the Fontan procedure is shown in Fig. 1. The SpO_2_ increased from the baseline of 84.6 ± 3.6% to the long-term value of 95.0 ± 2.5% (*p* = 0.07) (Fig. 1a). The CTR significantly decreased from the pre-BCPS time point to the long-term follow-up time point after the Fontan procedure (55.9 ± 7.0% vs. 41.9 ± 5.9%; *p* ≤ 0.001) (Fig. 1b). There was a continuous decrease of SVEDP with a baseline significantly higher than SVEDP at pre-Fontan, post-Fontan, and long-term-Fontan time points (11.4 ± 3.2 vs. 8.2 ± 3.0, 7.7 ± 2.2, and 6.9 ± 3.6, respectively; *p* < 0.05) (Fig. 1c). There was no significant decrease of PAI between pre-BCPS and pre-Fontan time points (494.7 ± 282.6 vs. 485.1 ± 272.3; *p* = 0.82). Long-term follow-up PAI after the Fontan procedure was significantly lower than that at pre-BCPS time point (269.5 ± 100.5 vs. 494.7 ± 282.6; *p* = 0.002) (Fig. 1d). The mean PAP and Rp at the long-term follow-up after the Fontan procedure were 11.1 ± 3.8 and 1.9 ± 1.4, respectively, and no significant changes were observed during the study period (Fig. 1e, f).

Laboratory data are summarized in Table 3. There was no significant change in the BNP levels during the study period; the BNP level was 36 ± 45 pg/mL at the long-term follow-up time point (116 months after the Fontan). Although AST, ALT, γ-GTP, and T-Bil. levels gradually increased, they were within the normal range (34.8 ± 7.0 U/L, 32.1 ± 11.1 U/L, 52.3 ± 23.6 U/L, and 0.8 ± 0.4 mg/dL, respectively) at the long-term follow-up time point. Type IV collagen, hyaluronic acid, and procollagen levels gradually increased and exceeded the normal range (11.9 ± 3.6 ng/mL, 66.8 ± 26.0 ng/mL, and 5.5 ± 11.7 U/mL, respectively) at the long-term follow-up time points.

**Table 3:** Laboratory data of the evaluated brain natriuretic peptide (BNP) and liver function and fibrosis marker levels

	Normal value	1 month after the Fontan (*n* = 22)	Mid-term (54 ± 18 months after the Fontan) (*n* = 13)	Long-term (116 ± 7 months after the Fontan) (*n* = 8)
BNP	0–18 pg/mL		41 ± 98	36 ± 45
AST	13–30 U/L	33.6 ± 5.6	36.6 ± 9.4	34.8 ± 7.0
ALT	10–42 U/L	17.5 ± 7.4	24.3 ± 7.8	32.1 ± 11.1
γGTP	13–64 U/L	38.1 ± 24.8	49.4 ± 14.4	52.3 ± 23.6
T-Bil	0.4–1.5 mg/dL	0.6 ± 0.3	0.7 ± 0.4	0.8 ± 0.4
Type IV collagen	0–6 ng/ mL		10.1 ± 2.6	11.9 ± 3.6
Hyauronic acid	0–50 ng/mL		44.8 ± 28.0	66.8 ± 26.0
Procollagen	0.3–0.8 U/mL		1.4 ± 0.4	5.5 ± 11.7

*AST* Aspartate aminotransferase, *ALT* Alanine aminotransferase, *γ-GTP* Gamma glutamic transpeptidase, *T-Bil* Total bilirubin

## Discussion

Our results suggest that APBF at BCPS helps maintain the Fontan circulation with high PAI, low PAP, low Rp, and low SVEDP without any major adverse event at the long-term follow-up after the Fontan procedure. However, our results also indicate that despite good Fontan conditions, liver fibrosis markers increase after the Fontan procedure.

BCPS can reduce cardiac volume overload and PAP [2, 15]. However, BCPS allows reduced pulmonary blood flow compared with normal circulation, leading to limited PA growth before Fontan completion (5). Moreover, PA growth after Fontan completion is reduced compared with somatic growth, and this phenomenon may disturb optimal long-term Fontan circulation [12]. Although APBF can preserve the potential of PA growth, excessive pulmonary blood flow could cause ventricular volume overload, atrioventricular valve regurgitation, and PAP elevation [16–18]. To avoid these concerns, Yoshida and colleagues developed a surgical management using an appropriately adjusted ABPF and reported its positive outcome compared with uncontrolled ABPF [19]. We reported a similar result when maximum APBF was regulated by a 3.5–4.0-mm SP shunt or retightened antegrade PA flow in our series [20]. After BCPS, the patients in the present series also showed good PA and ventricular condition throughout the follow-up.

Although PA and ventricular conditions are important factors for Fontan completion and its late outcome, some controversies remain. Lehner and colleagues reported that reduced PA diameters measured using the McGoon ratio and PAI did not adversely affect the early outcome of Fontan palliation [21]. However, Chowdhury and colleagues reported in their histomorphometric analysis that low PAI is significantly associated with the presence of severe intimal lesions, thrombus, abnormal smooth muscle extension, a reduced mean-indexed area of the intrapulmonary arteries, and poor postoperative outcome [22]. Hosein and colleagues [23] reported that preoperatively impaired ventricular function and elevated PAP have an adverse impact on both early and late outcomes. Ovroutski and colleagues reported that small PAs and low PAI are associated with elevated PAP as a late outcome after the Fontan procedure, with a correlation between low PAI and unfavorable late outcome [24]. Altogether, we believe that APBF is effective in Fontan circulation improvement based on our present results. Unfortunately, little data is available on the effects of BCPS with APBF on the Fontan circulation. Sugimoto and colleagues reported that patients who underwent BCPS with APBF had better-developed PA prior to the Fontan procedure, shorter pleural drainage duration, and shorter hospital stay after the Fontan procedure [25]. In our study, PA developed well and PAI was maintained at 485.1 ± 272.3 with low mean PAP (11.3 ± 3.8 mmHg) and Rp (1.1 ± 0.6 units) before the Fontan procedure. SVEDP decreased from 11.4 ± 3.2 to 8.2 ± 3.0 mmHg during the interstage of the Fontan procedure. In comparison with other reports (10,16,21), PAP, Rp, and SVEDP were similar or lower, whereas PAI was superior in our study. There were no intrapulmonary shunts or atrioventricular valve regurgitations that required intervention by the Fontan procedure. Adjusted ABPF prevented the decrease in PAI without increasing PAP and ventricular volume overload before the Fontan procedure. These parameters were maintained at the mid-to-long-term follow-up, resulting in good Fontan circulation in all patients with NYHA status I. However, it is noteworthy that although several researchers have reported better catheter data in APBF cases, no evident difference in mid-term survival rate between APBF and non-APBF cases has been observed. Furthermore, Schreiber and colleagues [26] achieved good outcomes in non-APBF cases; accordingly, we think that the long-term clinical data of both APBF and non-APBF cases require further evaluation in future studies.

Our results show that even in good Fontan condition, liver fibrosis markers increase after the Fontan procedure. The mechanism underlying Fontan-associated liver dysfunction is different from that of other liver diseases such as viral or alcoholic hepatitis. The evaluation of liver function is a challenge and there are various opinions on it. Nakano and colleagues reported that despite normal AST and ALT levels, a substantial number of patients show abnormally elevated levels of P-III-NP and collagen type IV, which are sensitive serum markers for liver fibrosis [27]. In this report, the mean CVP and SVEDP were 10 mmHg and 7.7 mmHg, respectively, at > 10 years after the Fontan procedure. Although these data support good Fontan condition at the mid-to-long-term follow-up, the liver fibrosis markers are elevated. In our study, collagen type IV, procollagen, and hyaluronic acid levels were elevated at the mid-to-long-term follow-up, although PAP and Rp were low, similar to the report by Nakano. Conversely, Wu et al. argued that the initial high levels of liver function markers, such as hyaluronic acid levels, may be unsuitable in Fontan patients because they did not correlate with the degree of hepatic fibrosis and did not predict cirrhosis in their research [28]. Further studies are necessary to evaluate liver dysfunction in Fontan patients at the long-term follow-up. We intend to examine the liver function and echo findings to clarify the effects of APBF on Fontan circulation during long-term follow-up. It is also important to develop surgical strategies to obtain reduced CVP after the Fontan procedure as much as possible.

The limitations of this study include the small size of the patient population, the homogeneity of the study group, and its retrospective nature. We did not compare the APBF cases with non-ABPF cases. In the future, larger cohorts and long-term studies must clarify whether APBF should be preserved at BCPS when considering the long-term prognosis of patients undergoing the Fontan procedure.

## Conclusion

In conclusion, the fine adjustment of APBF during BCPS can be useful for suppressing the reduction in PA size before the Fontan procedure, particularly in patients with underdeveloped PA. At the mid-to-long-term follow-up, no patient experienced any adverse event, and all patients showed NYHA status I. Even when Fontan circulation is well-maintained during follow-up, liver fibrosis marker levels can be elevated.

## Supplementary information

**Additional file 1: Supplementary Table 1**. The diagnosis and type of dominant ventricle of the patients.

## Data Availability

All data generated or analyzed during this study are included in this published article [and its supplementary information files].

## Abbreviations

BCPS: Bidirectional cavopulmonary shunt
APBF: Additional pulmonary blood flow
CVP: Central venous pressure
SP: Systemic-to-pulmonary
PAB: Pulmonary artery banding
SVC: Superior vena cava
CPB: Cardiopulmonary bypass
PAI: PA index
PAP: Mean PA pressure
Rp: PA resistance
SVEDP: Systemic ventricular end-diastolic pressure
CTR: Cardiothoracic ratio
SpO2: Saturation of percutaneous oxygen
BNP: Brain natriuretic peptide
AST: Aspartate aminotransferase
ALT: Alanine aminotransferase
γ-GTP: Gamma-glutamyl transpeptidase
NYHA: New York Heart Association
