# Supplementary material for: Long-term results of additional pulmonary blood flow with bidirectional cavopulmonary shunt
Source: J Cardiothorac Surg. 2020 Sep 29;15:279. doi: 10.1186/s13019-020-01335-4 (PMC7526092; doi:10.1186/s13019-020-01335-4)
Supplement: Supplementary file 1 — Additional file 1: Supplementary Table 1. The diagnosis and type of dominant ventricle of the patients. [file 13019_2020_1335_MOESM1_ESM.docx]

Supplementary table 1: The diagnosis and type of dominant ventricle of the patients

| Characteristics | Number of patients |
| --- | --- |
| Primary diagnosis |  |
| DORV | 6 |
| TA | 4 |
| UVH | 4 |
| TGAIII | 3 |
| Corrected TGA | 3 |
| Others | 3 |
| Dominant ventricle | |
| Right | 7 |
| Left | 15 |
| Heterotaxy | 4 |

DORV: double outlet right ventricle; TA: tricuspid atresia; UVH: univentricular heart; TGA: transposition grate arteries
